# Evaluation of the relationship between serum estradiol levels on human chorionic gonadotropin administration day and intracytoplasmic sperm injection outcomes: A retrospective population-based study

**DOI:** 10.18502/ijrm.v19i7.9470

**Published:** 2021-08-16

**Authors:** Masoumeh Hajshafiha, Sima Oshnouei, Mahdieh Mostafavi, Sina Dindarian, Nazila Kiarang, Sedra Mohammadi

**Affiliations:** ^1^Department of Obstetrics and Gynecology, Urmia Reproductive Health Research Center, Urmia University of Medical Sciences, Urmia, Iran.; ^2^Clinical Research Development Unit, Imam Khomeini Hospital, Urmia University of Medical Sciences, Urmia, Iran.; ^3^Student Research Committee, Urmia University of Medical Sciences, Urmia, Iran.

**Keywords:** Intracytoplasmic sperm injection, In vitro fertilization, Estradiol, Pregnancy.

## Abstract

**Background:**

The correlation between high estradiol (E2) levels induced by controlled ovarian hyperstimulation (COH) and pregnancy is controversial.

**Objective:**

To assess the effect of serum E2 levels on the day of human chorionic gonadotropin administration on the intracytoplasmic sperm injection (ICSI) outcome.

**Materials and Methods:**

The current study included 551 participants who had undergone ICSI between May 2014 and May 2016. Based on E2 levels, the individuals aged < 37 yr (n = 502) and 37-42 yr (n = 49) were categorized into six and three groups, respectively. ICSI outcomes including the number of retrieved oocytes (NRO), number of embryos (NE), pregnancy rate, and abortion rate were analyzed in both groups.

**Results:**

Among participants aged < 37 yr, the NRO and NE were 8.69 ± 3.82 and 5.24 ± 2.32, respectively and they had a significant correlation with the E2 level on human chorionic gonadotropin administration day (p < 0.001 for both). Among participants aged > 37 yr, NRO and NE were 5.18 ± 3.17 and 3.40 ± 2.23, respectively, and the NRO (p < 0.001), NE (p < 0.001), pregnancy rate (p < 0.001), abortion rate (p = 0.007), and the number of grade A and B embryos (p = 0.003) had a significant association with the E2 level.

**Conclusion:**

COH is a costly procedure that may have negative effects on endometrial receptivity. Thus, in order to prevent these effects and also to reduce the costs of COH, we recommend gaining optimum number of oocytes rather than maximum number during the procedure.

## 1. Introduction

For more than 30 yr, in vitro fertilization (IVF) has been clinically used for the treatment of infertility (1). Transferring morphologically adequate embryos in the uterus by retrieving sufficient cumulus-oocyte complexes following an ovarian stimulation is essential for achieving pregnancy in human IVF (2). According to previous studies, the number of retrieved oocytes (NRO) has a positive impact on live birth rate. Consequently, obtaining mature follicles with good-quality oocytes determines an IVF-intracytoplasmic sperm injection (ICSI) procedure outcome (3, 4). Lately, in order to gain numerous follicles, ovulation induction methods such as gonadotropins and gonadotropin-releasing hormone analogues administration, and controlled ovarian hyperstimulation (COH) are being used. Playing a key role in the success rate of IVF, COH is an applicable way for achieving high-quality oocytes (5). Although COH increases the number of oocytes, it is accompanied by supraphysiological serum estradiol (E2) levels (2). Studies show controversial reports on the correlation of high E2 levels with the COH and pregnancy achievement. While some studies have reported a positive correlation (6), others have reported negative (7) or lack of correlation (8, 9). Also, the most important adverse effects of supraphysiological serum E2 levels are issues regarding oocyte and embryo quality, endometrial receptivity, and birth weight (10–12).

In this study, we aimed to evaluate the relationship between serum estradiol levels on human chorionic gonadotropin (hCG) administration day and ICSI outcomes.

## 2. Materials and Methods

In this retrospective population-based study, 551 participants who had undergone an ICSI procedure between May 2014 and May 2016 were enrolled. The demographic information of the participants was obtained via their medical records at the Reproductive Health Research Center, Urmia University of Medical Sciences, Urmia, Iran. All participants had undergone COH and their E2 levels were measured on the day of hCG administration. Based on the individuals' serum E2 levels on the day of hCG administration, those aged < 37 yr old (n = 502) were divided into six groups: group I (≤ 1500 pg/ml, n = 105), group II (1501–3000 pg/ml, n = 145), group III (3001–4500 pg/ml, n = 72), group IV (4501–6000 pg/ml, n = 47), group V (6001–7500 pg/ml, n = 29), and group VI (> 7500 pg/ml, n = 104). Likewise, considering the small number of participants aged 37–42 yr (n = 49), they were divided into three groups: group A (≤ 1500 pg/ml, n = 12), group B (1501–3000 pg/ml, n = 19), and group C (> 3000 pg/ml, n = 18). However, participants with a history of smoking, salpingectomy due to hydrosalpinx, ovarian cysts, endometriosis, those aged > 42 yr, and those with unrecorded E2 levels on hCG administration day or with follicle-stimulating hormone (FSH) levels >10 IU/mL were excluded from the study.

The administration of Suprefact (0.5 mg subdermal, Hoechst, Germany) was started on the 21${}^{\mathrm{st}}$ day of the previous menstrual cycle for individuals under treatment with long protocol. Then, on the second day of the menstrual cycle, FSH was administered. The FSH dose was adjusted according to the participants' age, serum FSH level and serum E2 level on the third day of their period, response of ovary to the drug in previous periods, ovary volume, and basal antral follicle count in ultrasonography. After starting the administration of gonadotropin, the suprefact dose was reduced by half and continued until the hCG administration day. The participants were followed using serial ultrasonography. When at least two follicles reached 17–18 mm of diameter, 5000–10,000 units of hCG (Pregnyl, MERCK, Germany) was administered. Using the standard protocols of ICSI, we retrieved the oocytes after about 14–36 hr and inseminated the retrieved oocytes. On oocyte retrieval day, we started administration of 100 mg of intramuscular progesterone in oil (Aburaihan Co., Tehran, Iran) for the participants in order to gain luteal support. Then after 72 hr, we transferred the embryos following follicular aspiration. Pregnancy was determined using the measurement of the serum hCG level 14 days after the embryo transfer. The ICSI outcomes including the NRO, the number of embryos (NE), the pregnancy rate (PR), and the abortion rate (AR) were assessed.

### Ethical considerations

All procedures were approved and performed in accordance with the ethical standards of the Ethics Committee of Urmia University of Medical Sciences, Urmia, Iran (Ethics code: IR.UMSU.REC.1394.104) and the 1964 Declaration of Helsinki and its later amendments or comparable ethical standards. Also, an informed consent was obtained from all participants prior to the study.

### Statistical analysis

Descriptive data are presented as mean ± standard deviation (SD) for each group. The distribution normality was assessed using the Kolmogorov–Smirnov test. For categorical variables, Chi-square test and Fisher's exact test were used and for comparing continuous variables among the groups, the one-way analysis of variance (ANOVA) and Kruskal–Wallis test were used. Statistical analysis was performed using the IBM SPSS Statistics 16.0 (SPSS Inc., Chicago, IL, USA). P-values < 0.05 were considered statistically significant.

## 3. Results

The mean age of all participants was 30.09 ± 5.31 yr. While the mean age of the individuals with E2 level ≤ 1500 was 30.35 ± 5.40 yr, the mean age of those with E2 levels 1501–3000 and 3001–4500 were 30.70 ± 5.24 and 29.96 ± 5.50 yr, respectively. Additionally, the participants with E2 levels 4501–6000 and 6001–7500 had the mean age of 29.35 ± 5.36 and 29.91 ± 5.67 yr, respectively. Finally, the mean age of the participants with E2 level > 7500 was 30.30 ± 4.70 yr. No significant correlation was seen between the age and E2 level (p = 0.76). Table I indicates the etiology of infertility in different estradiol groups. As seen in the table, male factor (48.8%) was the most common and adhesion and endometriosis (2.2%) was the least common etiology of infertility among the participants. According to Fisher's exact test, there was no significant difference among the different E2 groups according to the etiology of infertility (p = 0.19).

The ICSI outcomes including NRO, the NE, the PR, and the AR of the individuals aged < 37 and ≥ 37 yr are shown in Tables II and III, respectively. As seen in Table II, the NRO and NE in individuals aged < 37 yr were 8.69 ± 3.82 and 5.24 ± 2.32, respectively, while the PR and AR were 38.64% and 12.15%, respectively. Furthermore, the percentage of grade-A and -B embryos was 65.94%. Among the participants aged ≥ 37 yr, the NRO and NE were 5.18 ± 3.17 and 3.40 ± 2.23, respectively. Moreover, the PR and AR were 20.40% and 16.33%, respectively. Finally, the percentage of grade-A and -B embryos was 87.75%. Among the individuals aged < 37 yr, the highest NRO was observed in group VI and the lowest in group I. The correlation between the NRO and E2 level on hCG administration day was statistically considerable (p < 0.001; Kruskal–Wallis test). Furthermore, a significant correlation was seen between the NE and E2 level (p < 0.001; Kruskal–Wallis test). The highest NE was seen in group VI and the lowest in group I. This was caused by the highest and the lowest NRO in groups VI and I, respectively, rather than higher or lower fertilization rate. Group V had the lowest and group VI the highest PR. The association between the PR and E2 level was not significantly meaningful (p = 0.43; Chi-square test). Also, group IV had the lowest AR, while the highest AR was found in group V. The correlation between these two parameters was not significant (p = 0.09; Fisher's exact test). While the grade A and B embryos were mostly seen in group I, they were the least in group V. There was no significant correlation between the number of grade-A and -B embryos and E2 level (p = 0.17; Chi-square test).

According to Table III, among the participants aged ≥ 37 yr, group C had the highest and group A had the lowest NRO. The correlation between these two parameters was statistically significant (p < 0.001; Kruskal–Wallis test). The highest NE was also seen in group C and the lowest in group A. We observed a significant correlation between the E2 level and NE (p < 0.001; Kruskal–Wallis test). Additionally, there was a significant difference in the PR (p < 0.001; Fisher's exact test) and AR (p = 0.007; Fisher's exact test) among the groups. No pregnancy and abortion was seen in group B. The highest PR and AR were observed in group A. Finally, a significant association was observed between the number of grade-A and -B embryos and the E2 level in individuals aged ≥ 37 yr (p = 0.003; Fisher's exact test). All embryos in groups A and B were grade A and B. The lowest number of grade-A and -B embryos was observed in group C (Table III).

**Table 1:** Etiology of infertility in different estradiol groups among all participants


[1.455in,lr]**Etiology** **Estradiol **	**≤ 1500 (n = 117)**	**1501–3000 (n = 164)**	**3001–4500 (n = 77)**	**4501–6000 (n = 51)**	**6001–7500 (n = 32)**	**> 7500 (n = 110)**	**p-value**
**Male factor (n = 269)**	54 (20.1)	66 (24.5)	40 (14.9)	32 (11.9)	14 (5.2)	63 (23.4)
**Ovulation problem (n = 17)**	7 (41.2)	4 (23.5)	2 (11.8)	2 (11.8)	0 (0)	2 (11.8)	0.19${}^{*}$
**Tubal problem (n = 43)**	6 (13.9)	19 (44.2)	6 (13.9)	1 (2.3)	2 (4.6)	9 (20.9)	
**Adhesion and endometriosis (n = 12)**	3 (25)	4 (33.3)	1 (8.3)	2 (16.7)	1 (8.3)	1 (8.3)	
**Unexplained (n = 210)**	47 (22.4)	71 (33.8)	28 (13.3)	14 (6.7)	15 (7.1)	35 (16.7)	
Data presented as n (%),${}^{*}$Fisher's exact test							

**Table 2:** Outcomes of ICSI according to the E2 levels on the day of hCG administration in participants aged < 37 yr


[1.455in,lr]**Parameter** **Group **	**I (n = 105)**	**II (n = 145)**	**III (n = 72)**	**IV (n = 47)**	**V (n = 29)**	**VI (n = 104)**	**p-value**
**NRO${}^{\alpha }$**	5.60 ± 2.49	7.55 ± 2.85	9.36 ± 3.49	9.19 ± 3.42	11.10 ± 3.10	12.04 ± 3.44	< 0.001${}^{*}$
**NE${}^{\alpha }$**	3.40 ± 1.25	4.57 ± 1.77	6.01 ± 2.32	5.51 ± 1.83	5.93 ± 2.12	7.18 ± 2.29	< 0.001${}^{*}$
**PR${}^{\beta }$**	35.2%	40.68%	37.5%	38.2%	27.5%	43.27%	0.43${}^{**}$
**AR${}^{\beta }$**	16.2%	8.28%	15.28%	6.38%	37.93%	6.73%	0.09${}^{***}$
**Grade-A and -B embryos${}^{\beta }$**	74.28%	63.47%	69.44%	65.96%	58.62%	60.57%	0.17${}^{**}$
${}^{\alpha }$Data presented as Mean ± SD, ${}^{\beta }$Data presented as %, ${}^{*}$Kruskal–Wallis test, ${}^{**}$Chi-square test, ${}^{***}$Fisher's exact test, NRO: Number of retrieved oocytes, NE: Number of embryos, PR: Pregnancy rate, AR: Abortion rate							

**Table 3:** Outcomes of ICSI according to the E2 levels on the day of hCG administration in participants aged ≥ 37 yr


[1.455in,lr]**Parameter** **Group **	**A (n = 12)**	**B (n = 19)**	**C (n = 18)**	**p-value**
**NRO${}^{\alpha }$**	3.08 ± 0.67	4.95 ± 0.78	6.83 ± 3.03	< 0.001${}^{*}$
**NE${}^{\alpha }$**	2.08 ± 0.29	3.16 ± 0.37	4.56 ± 1.42	< 0.001${}^{*}$
**PR${}^{\beta }$**	66.7%	0%	11.1%	< 0.001${}^{**}$
**AR${}^{\beta }$**	41.7%	0%	16.7%	0.007${}^{**}$
**Grade-A and -B embryos${}^{\beta }$**	100%	100%	66.7%	0.003${}^{**}$
${}^{\alpha }$Data presented as Mean ± SD, ${}^{\beta }$Data presented as %, ${}^{*}$Kruskal–Wallis test, ${}^{**}$Fisher's exact test, NRO: Number of retrieved oocytes, NE: Number of embryos, PR: Pregnancy rate, AR: Abortion rate				

## 4. Discussion

In the current study, we aimed to assess the serum levels of E2 on the hCG administration day and assess its effect on an ICSI outcome. Several studies have suggested that high serum E2 concentration on hCG administration day has a positive effect on pregnancy, however, others have reported contrary to this (2, 13). The results of the current study showed that the mean NRO increased gradually as the serum E2 levels increased up to 7500 pg/ml in women aged < 37 (p < 0.001) and ≥ 37 (p < 0.001) yr. We also observed a significantly higher mean NRO in the group with serum E2 levels > 7500 pg/ml compared to other groups. This finding was also observed in the study conducted by Pena and co-authors who reported that NRO increased continuously when serum E2 levels exceeded 3000 pg/ml (14). Also, another study showed that significantly higher numbers of oocytes were retrieved in the group with serum E2 levels > 4000 pg/ml (15.3 ± 6.6) (3).

Moreover, the result of previous studies showed that the supraphysiological levels of E2 have a disadvantageous impact on the outcome of IVF in individuals aged > 35 yr. It has been described that the effect of serum E2 level on hCG administration day on NRO and PR depends on the age of the women (3, 15). In the current study, PR was higher in increased concentrations of serum E2 level (≥ 7500 pg/ml) in women aged < 37 yr. Conversely, in women aged ≥ 37 yr, PR was higher among those with low-serum E2 levels (< 1500 pg/ml). Some studies reported no significant differences in the implantation and PR among E2 groups on the day of ovulation trigger (16). It was concluded that high E2 levels in assisted reproductive technology (ART) cycles can cause gland-stromal dyssynchrony and impair implantation (17). However, in some studies, a positive correlation between an increased level of E2 and higher PR have been reported (6). Similar results have been reported that PR increase progressively with increasing levels of peak serum E2 but they suggested that there is an optimal range of serum E2 level influencing the IVF-ET outcome and that the maximum NRO does not always indicate higher PR (3, 6). These findings state that even significantly higher NRO does not always indicate higher PR and the selection of desired number of good-quality embryos to transfer is a key to successful IVF procedure (18).

We found that NE was strongly correlated with higher serum levels of E2 and the differences were statistically significant both in women aged < 37 and ≥ 37 yr. Also, a significant difference was observed between the number of good- and poor-quality embryos in women aged ≥ 37 yr. However, this difference was not significant in women aged < 37 yr. A prospective study was conducted on 207 subjects and a significantly higher total embryo score (12 ± 12.6) of the embryos in the 75${}^{\mathrm{th}}$ centile E2 group (> 2446 pg/ml) than the 25${}^{\mathrm{th}}$ and 50${}^{\mathrm{th}}$ centile groups was observed (16). Another study also found no effect of E${}_{2}$ on mature follicle on the day of hCG and the number of obtained grade-A embryos (19). In another study, the rate of good-quality embryos did not increase progressively with increasing levels of peak serum E2 and also higher levels of serum E2 did not affect the number of good-quality embryos (5). Our results were in line with the aforementioned study in women aged < 37 yr. However, in women aged ≥ 37 yr, the rate of good-quality embryos was significantly higher in those with lower E2 levels. Moreover, we demonstrated that AR did not increase with increasing serum E2 levels in both younger and older women. However, there was a significant difference in the AR among the different E2 groups in women older than 37 yr. The assessment of the effect of increased E2 levels on the day of hCG administration on birth outcomes revealed that there was not a considerable difference among the E2 groups in AR and good-quality embryo rate. On the other hand, the study of Wang et al. (5) on singleton pregnancies in high-E2 (E2 ≥ 3757 pg/mL) group indicated a considerable elevation in AR in individuals older than 37 yr. We recommend further studies especially prospective ones with larger sample sizes in order to assess the correlation between the AR and serum E2 level. The strength of our study was the large sample size in participants below the ages of 37 yr. But unfortunately, due to lower number of individuals aged 37–42 yr, the results gained for these participants cannot be generalized to all, and further studies are recommended for this age group.

COH is apparently a key factor in performing IVF-ICSI successfully, as it improves the chance of fertilization and PR. On the other hand, it inhibits embryo implantation after IVF by decreasing the endometrial receptivity. This method may result in supraphysiological E2 levels and inevitably causes ovarian hyperstimulation syndrome during the luteal phase. Each of these follicles can lead to high E2 production – almost 10 times or more than those found during spontaneous cycles (3, 20). The reason for low implantation rate in IVF-ICSI method might be the disturbance in the quality of transferred embryos and endometrial receptivity even with the transfer of apparently healthy embryos (21). Thus, it can be concluded that an attempt to gain maximum number of oocytes during COH may affect pregnancy in a negative way.

## 5. Conclusion

In conclusion, our findings suggest that an increased level of E2 does increases the NRO but it has no effect on number of transferred embryos and PR. High levels of E2 may have negative effects on endometrial receptivity. Thus, for preventing the adverse effects of COH on endometrial receptivity and also in order to decrease the costs of the procedure, gaining optimum number of oocytes should be the aim of COH rather than gaining maximum number.

##  Conflict of Interest

The authors declare no conflict of interest.
